# Neuroprotective mechanism of low-dose sodium nitrite in oxygen-glucose deprivation model of cerebral ischemic stroke in PC12 cells

**DOI:** 10.17179/excli2018-1947

**Published:** 2019-04-08

**Authors:** Nader Shakib, Mohammad Hassan Khadem Ansari, Pouran Karimi, Yousef Rasmi

**Affiliations:** 1 Department of Biochemistry, Faculty of Medicine, Urmia University of Medical Sciences, Urmia, Iran; 2 Neurosciences Research Center (NSRC), Tabriz University of Medical Sciences, Tabriz, Iran

**Keywords:** sodium nitrite, oxygen and glucose deprivation, PC12 cells, endoplasmic reticulum stress, Ca2+ release

## Abstract

The purpose of this study was to clarify the mechanisms of the protective effects of low-dose sodium nitrite (SN) on oxygen and glucose deprivation (OGD)-induced endoplasmic reticulum (ER) stress in PC12 cells. The PC12 cells were exposed to 4 h of OGD and treated with 100 μmol SN. The expression and activity of ER stress markers, including PKR-like endoplasmic reticulum kinase (PERK), transcription factor 6 (ATF6), CCAAT/enhancer binding protein homologous protein (CHOP), as well as caspase-12 and -3, were detected by immunoblotting assay. Fluorescence staining was used to detect the levels of reactive oxygen species (ROS) and Ca^2+^ release from the ER. Cell viability was also evaluated by MTT assay. It was found that SN significantly inhibited ROS production and Ca^2+^ release from the ER in OGD-injured PC12 cells. Moreover, ER stress marker expression and cleaved fragments of caspase-3 and -12 in OGD-injured PC12 cells were decreased after SN treatment. These findings were accompanied by a significant increase in cell viability. It seems that SN exerts a neuroprotective effect at least partially through reduction of ROS-mediated ER stress caused by OGD insult.

## Introduction

According to World Health Organization statistics, stroke is the second leading cause of death worldwide after myocardial infarction (Moskowitz et al., 2010[54]). Recent developments have shown that endoplasmic reticulum (ER) stress is an important cause of neuronal injury following cerebral ischemia (Anelli and Sitia, 2008[4]; Ma and Hendershot, 2004[47]; Nakka et al., 2010[57]). Neurons are vulnerable to ischemia and disturbance in the central nervous system leads to severe effects such as paresis, memory disruption and even neuronal death (Anderson and Arciniegas, 2010[3]). Hypoxia and hypoglycemia, two common features in ischemic stroke, increase the generation of reactive oxygen species (ROS), cellular membrane depolarization, and glutamate release from neurons. Moreover, in neuronal cells, ischemia increases the release of Ca^2+^ from the ER to the cytosol, promoting excitotoxicity and apoptosis (Ankarcrona et al., 1995[5]; Lipton and Rosenberg, 1994[46]; Pellegrini-Giampietro et al., 1997[67]). 

The ER is the main organelle for secretory pathways in all eukaryotic cells. Neuronal cells in particular have a highly developed ER. This organelle contributes to protein folding, biosynthesis, translocation, and post-translational modifications including glycosylation, disulfide bond formation and chaperone-mediated protein folding processes, which are essential for effective functioning. ER also serves as a cellular Ca^2+^ reservoir (Chang et al., 2006[15]; Hampton, 2002[25]; Schroder and Kaufman, 2005[75]). During brain ischemia, neuronal cells endure lack of the oxygen and glucose required for ATP synthesis, which results in a disturbance in energy consuming protein-folding and the accumulation of unfolded proteins (UPs) in the ER (DeGracia et al., 2002[18]). Accumulated UPs increase the size of ER membranes, accelerate degradation of UPs, increase the translation of folding chaperones, and inhibit synthesis of other proteins, which as a whole is denoted as ER stress (Herrmann et al., 2013[32]; Kaufman, 1999[36]; Kaufman et al., 2002[37]; Oyadomari et al., 2002[61]).

In response to ER stress, three important sensor and transducer proteins on the ER membrane, PKR-like endoplasmic reticulum kinase (PERK), transcription factor 6 (ATF6), and inositol-requiring enzyme 1 (IRE1), are activated (Harding et al., 2000[26]; Haze et al., 1999[30]; Wang et al., 1998[84]). When cellular stress is very serious or prolonged, these components can promote apoptosis signaling pathways through the activation of the CCAAT/enhancer binding protein homologous protein (CHOP), c-Jun N-terminal kinase (JNK) and caspase-12 proteins (Ferri and Kroemer, 2001[23]; Paschen and Mengesdorf, 2005[66]; Szegezdi et al., 2006[78]). The induction of CHOP mRNA by brain ischemia or hypoxia has been demonstrated in previous studies to be one of the most significant pathways leading to neuronal apoptosis (Carmeliet et al., 1998[13]; Doutheil et al., 1997[20]; Jin et al., 2001[35]; Paschen et al., 1998[65]), CHOP can also reduce the expression of B-cell lymphoma-2 (Bcl-2) leading to ER stress and an increase in oxidative damage (Ferri and Kroemer, 2001[23]; Mori, 2000[52]). 

Caspase-12 is a member of the interleukin-1β converting enzyme (ICE) subfamily of caspases which normally exist in an inactive pro-enzyme state attached to the ER membrane (Badiola et al., 2011[8]; Nakagawa et al., 2000[56]). Active caspase-12 triggers caspase-9 and caspase-3 activation, resulting in DNA fragmentation. Caspase-12 activation can be induced by oxygen and glucose deprivation (OGD) in glial cells (Martinez et al., 2010[48]; Rao et al., 2001[73]). 

It has been reported that the overproduction of ROS is a substantial cause of cerebral injury following ischemia/reperfusion (Qi et al., 2016[71]). Mitochondria which are damaged during prolonged ischemic insult are the predominant source of ROS. ROS generation initiates several intracellular signaling cascades and concomitantly induces mitochondrial dysfunction and ER stress, which activates apoptosis (Hildeman et al., 2003[33]; Ray et al., 2012[74]; Tajiri et al., 2004[79]). 

Nitrite, a physiological reservoir of nitric oxide (NO), is a powerful mediator of cytoprotection following ischemia/reperfusion which acts by reducing mitochondrial ROS generation. Moreover, NO-derived from NO donors (NODs) has a scavenging effect on ROS and directly reacts with ROS or regulates mitochondrial ROS levels by mitochondrial complex I modification (Pluta et al., 2001[68]; Raat et al., 2009[72]; Steiner et al., 2002[77]). Nitrite is reduced to NO by the reductase system under ischemia/hypoxia conditions (Calvert and Lefer, 2010[12]). 

In the present study, the effect of sodium nitrite (SN) was investigated on ROS production, Ca^2+^ release and ER stress-related markers (phospho-PERK, ATF6, CHOP, caspase-12, and caspase-3) in OGD-exposed PC12 cells.

## Materials and Methods

### Chemicals and antibodies

The following antibodies (Santa Cruz Biologics; USA) were used in Western blotting: polyclonal rabbit antibodies against caspase-3 (1:500), caspase-12 (1:500), CHOP (1:500), p90ATF6 (1:500) and phospho-PERK (1:500). Nitrite sodium (Sigma; 563218) was a gift from Dr. Amiri. Fetal bovine serum (FBS), Dulbecco's Modified Eagle Medium (DMEM) and trypsin were obtained from Hyclone (USA). Sigma-Aldrich Fluorometric Intracellular ROS kits and green fluorescence (MAK143) were used for ROS staining and Goryo Chemical red fluorescent Ca^2+^ probe CaTM-3 AM (GC 501) was used to determine Ca^2+ ^release.

### Cell culture

The pheochromocytoma derived-PC12 cell line was purchased from Pasteur Institute (Iran), cultured in DMEM enriched with 10 % horse serum and incubated under normoxic conditions (95 % air and 5 % CO_2_) at 37 °C. The four study groups were: normal control (NC), normoxic/normoglycemic (NO/NG), OGD for 4 h (OGD), 100 µM SN added to the culture medium concomitantly with OGD onset (OGD+SN) and 100 µM SN added to NO/NG (NC+ SN). 

For induction of the *in vitro* OGD model, PC12 cells were cultured in six-well plates in the NO/NG condition. At 80 % confluence, the culture medium was replaced by glucose-free Hank's Balanced Salt Solution (HBSS) and incubated in a homemade hypoxia chamber (95 % N_2_, 5 % CO_2_) at humidity 60 % for 4 h. In the treatment groups, PC12 cells were cultured in the aforementioned media and 100 µM SN was applied to the medium in the presence or absence of OGD. 

### ROS level detection

ROS or its intermediates were produced by the uncompleted reduction of oxygen. Organisms living under aerobic conditions generate various kinds of ROS molecules, such as superoxide (• O_2_^−^), hydrogen peroxide (H_2_O_2_), hydroxyl radicals (OH^−^) and singlet oxygen. ROS are highly reactive molecules and are exceedingly unstable, so detection of ROS depends on measurement of the end products formed when they react with specific substances. ROS such as superoxide and hydrogen peroxide can be detected conventionally by staining techniques.

In the present study, 2',7'-dichlorodihydrofluorescein diacetate (DCFH-DA; Sigma; USA) was used to measure ROS levels. Briefly, PC12 cells were cultured in growth medium to reach 1 ⨯ 10^6 ^cells per well in a 96-well microplate and 100 μL/well DCFH-DA working solution was added directly to the medium and incubated at 37 °C for 15 min. The cells were then washed with phosphate buffered saline (PBS) once and kept on ice for immediate DCF detection. Fluorescent images were obtained under a fluorescence microscope (Olympus; Japan). Intracellular ROS generation was monitored by measuring the fluorescent intensity of the cells using a BioTek Synergy plate reader and the results were expressed as the percentage of control. The wavelength used for excitation was set at 490 nm and for emission was set at 525 nm.

### Immunoblotting assay

PC12 cells were homogenated in ice-pre cold RIPA lysis buffer containing protease inhibitors and centrifuged at 12000 g for 15 min at 4 °C. The supernatant was taken for quantification of protein concentration using the Bradford method. The samples then were mixed 1:1 with sample loading buffer 2X (Sigma; USA) and boiled for 10 min before loading onto 10 % SDS-polyacrylamide gel for electrophoresis. After transfer of the proteins onto the methanol pre-activated polyvinylidene fluoride (PVDF) membrane, the membranes were incubated with blocking solution for 2 h at room temperature. The membrane then was incubated overnight with polyclonal anti-ATF6, anti-CHOP, anti-caspase-3 and -12 and anti-β-actin antibodies at 4 °C. After washing three times with PBS, the membrane was incubated with an appropriate horseradish peroxidase conjugated anti-rabbit secondary antibody containing 0.1 % Tween-20 for 2 h at room temperature. The blots were detected using a chemiluminescence detection kit (Amersham Biosciences; USA) and radiographic film (Fuji; Japan). The density of the target bands was measured by Image J software. β-actin was used as a loading control (Farajdokht et al., 2018[21]). 

### Ca^2+ ^release 

Goryo Chemical CaTM-2 was used to determine the rate of Ca^2+ ^release. After seeding the cells in six-well plates, the treated and control cells were washed twice in Krebs buffer (pH 7.4) composed of 132 mM NaCl, 4 mM KCl, 1.4 mM MgCl_2_, 6 mM glucose, 10 mM HEPES, 10 mM NaHCO_3_ and 1 mM CaCl_2_. Then, 50 µg CaTM-2 was dissolved in 41 µL of DMSO to a concentration of 1 mM. To improve the induction efficiency and inhibit localization of the probe, Pluronic F-127 was added. Next, an aliquot of the stock solution was diluted to a final concentration of 1-10 µM in loading medium. The culture medium was removed from the wells and washed with loading medium. Stain solution then was added to the wells and incubated for 10-60 min at 37 °C and 5 % CO_2_. After staining, the stain solution was discarded and the wells were washed three times. The changes in intracellular fluorescence intensity were measured using a fluorimeter. The excitation wavelength was set at 597 nm and the maximum peak of the fluorescent wavelength was detected at 609 nm.

### MTT assay

PC12 cells were seeded in 96-well plates at an initial density of 10^4^ cells/100 μl/well. After 48 h, when cell count reached 10^5^, cell viability was determined using the 3-[4,5-dimethylthiazol-2-yl]-2,5-diphenyl-tetrazolium bromide (MTT) assay. At the end of the incubation period (4 h), the supernatant was removed and 200 µl of DMSO was added to each well. Absorbance was read at 570 nm using a microplate reader (Stat fax; Awareness; USA) and the results were expressed as percentage of control.

### Statistical analysis

The data are expressed as mean ± SEM. The comparisons of means were done by one-way analysis of variance (ANOVA) followed by Tukey's post-hoc test (SPSS, version 20; USA). A p-value of <0.05 was considered statistically significant. The statistical power was 0.8 at α = 0.05 and β = 0.2. 

## Results

### Effect of SN on ER stress indicators in OGD-injured PC12 cells

**Figure 1:**
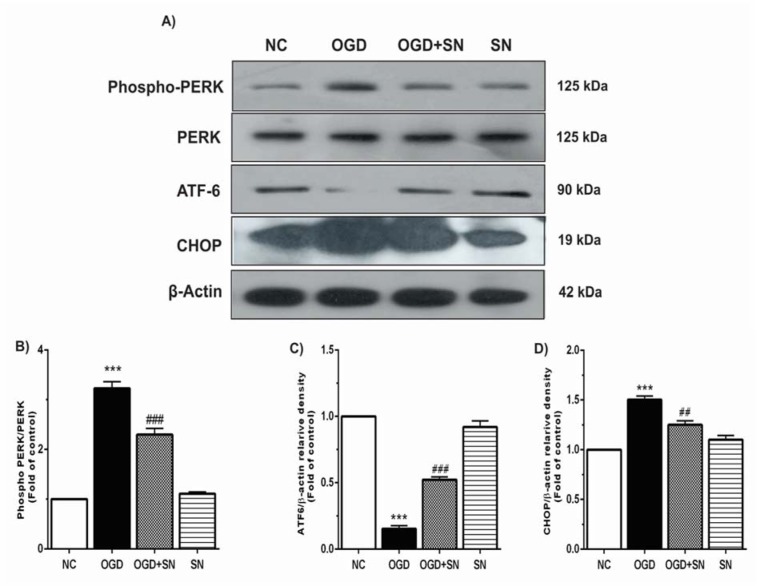
SN up-regulated ATF6 and down-regulated CHOP and PERK in OGD-injured PC12 cells: (A) immunoblotting images of expression of p-PERK, PERK, ATF6, CHOP and β-actin proteins under different experimental conditions; (B), (C), and (D), respectively, quantitative densitometric analysis of the p-PERK, ATF6 and CHOP protein bands. Data are shown as means ± SEM (n=3): ^***^p<0.001 vs. NC; ^###^p<0.01 vs. OGD [NC: normal control; OGD: oxygen and glucose deprivation (4 h); OGD-SN: oxygen and glucose deprivation (4 h) co-treated with SN (100 µm); SN: sodium nitrite (100 µm) treated].

The phosphorylation of PERK (p-PERK) and expressions of ATF6 and CHOP as the common indicators of ER stress were determined by immunoblotting technique under four experimental conditions. The results showed that OGD significantly increased immunoreactivity of p-PERK (p<0.001; Figure 1B(Fig. 1)) and CHOP (p<0.001; Figure 1D(Fig. 1)) as compared to the NC, which decreased after the SN treatment. Moreover, OGD considerably decreased ATF6 expression (p<0.001; Figure 1C(Fig. 1)) as compared to the NC condition. However, parallel SN treatment with OGD onset significantly increased ATF6 expression (p<0.001) in PC12 cells.

### SN decreased cleavage of Caspase-3 and Caspase-12 in OGD-treated PC12 cells 

**Figure 2:**
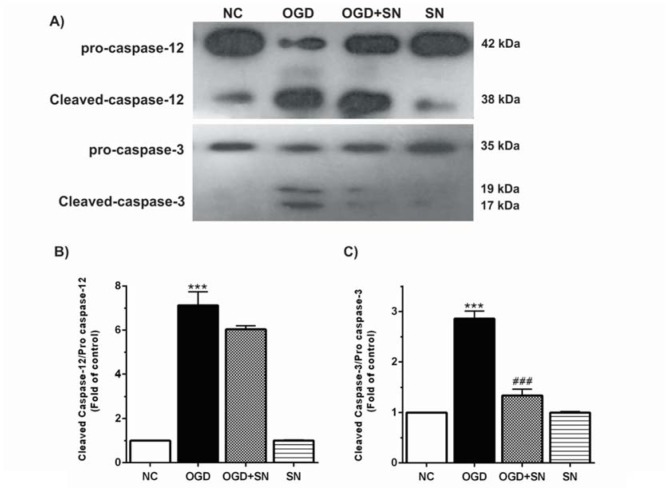
SN down-regulated cleavage of caspase-12 and caspase-3 in OGD injured PC12 cells: (A) immunoblotting images of cleaved caspase-12, procaspase-12, cleaved caspase-3, and procaspase-3 in different experimental conditions; (B) and (C), respectively, quantitative densitometric analysis of cleaved caspase-12/procaspase-12 and cleaved caspase-3/procaspase-3 ratios. Data are shown as means ± SEM (n=3): ^***^p<0.001 vs. NC; ^###^p<0.01 vs. OGD [NC: normal control; OGD: oxygen and glucose deprivation (4 h); OGD-SN: oxygen and glucose deprivation (4 h) co-treated with SN (100 µm); SN: sodium nitrite (100 µm) treated].

The results also demonstrate that OGD markedly increased the ratio of cleaved caspase-12/procaspase-12 (p<0.001; Figure 2B(Fig. 2)) and cleaved caspase-3/procaspase-3 (p<0.001; Figure 2C(Fig. 2)) as compared to the NC condition. On the other hand, cleaved caspase-3/procaspase-3 ratio was significantly decreased in the OGD-SN condition (p<0.001). 

### SN decreased ROS production in OGD-treated PC12 cells

**Figure 3:**
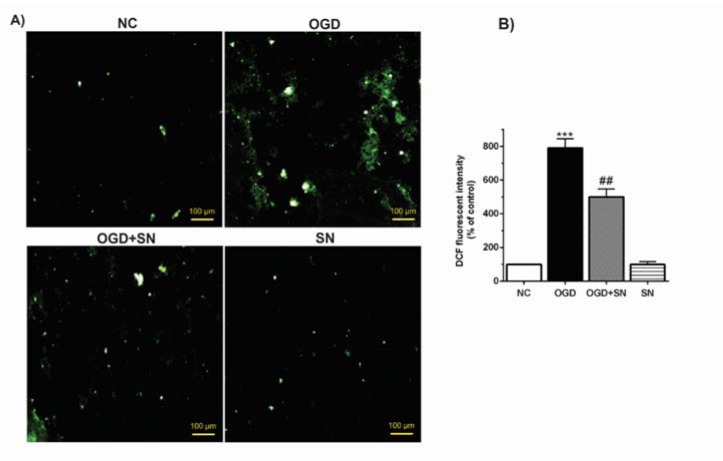
SN attenuated ROS levels in OGD-injured PC12 cells: (A) fluorescence emission spectrum of DCF probe in PC12 cells exposed to 4 h OGD and SN 100 µM treatment under different experimental conditions; (B) percentage of relative intensity of DCF fluorescence per mg protein (n=3): ^***^p< 0.001 vs. NC; ^##^p<0.01 vs. OGD [NC: normal control; OGD: oxygen and glucose deprivation (4 h); OGD-SN: oxygen and glucose deprivation (4 h) co-treated with SN (100 µm); SN: sodium nitrite (100 µm) treated].

Figure 3(Fig. 3) shows that the percentage of DCF fluorescence intensity as an index of the rate of ROS production increased significantly (p<0.001) following 4 h of OGD in PC12 cells. Nevertheless, in the OGD+SN condition, co-treatment with SN (100 µM) significantly decreased ROS generation levels (p<0.01) as compared to the OGD condition (Figure 3(Fig. 3)).

### SN decreased intracellular Ca^2+^ release from ER

**Figure 4:**
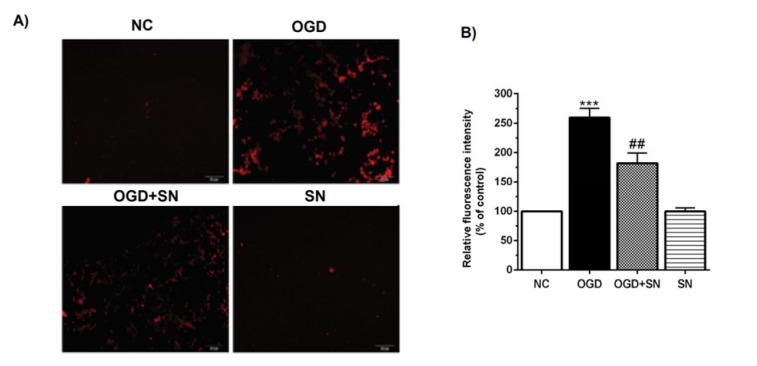
SN decreased Ca^2+^ release from ER in OGD-exposed PC12 cells: (A) intracellular Ca^2+ ^levels stained by the Fluo4-AM dye; (B) relative fluorescence intensity analysis of intracellular Ca^2+^ under different experimental conditions (n=3): ^***^p<0.001 vs. N; ^##^p<0.01 vs. OGD [NC: normal control; OGD: oxygen and glucose deprivation (4 h); OGD-SN: oxygen and glucose deprivation (4 h) co-treated with SN (100 µm); SN: sodium nitrite (100 µm) treated].

Figure 4(Fig. 4) shows that the lowest release of Ca^2+^ was observed in the NC and SN conditions. In the OGD condition the release of Ca^2+ ^increased considerably (p<0.001). Co-treatment of the PC12 cells with 100 µM SN in the OGD+SN condition significantly decreased Ca^2+^ release (p<0.01).

### SN increased cell viability in OGD-treated PC12 cells

**Figure 5:**
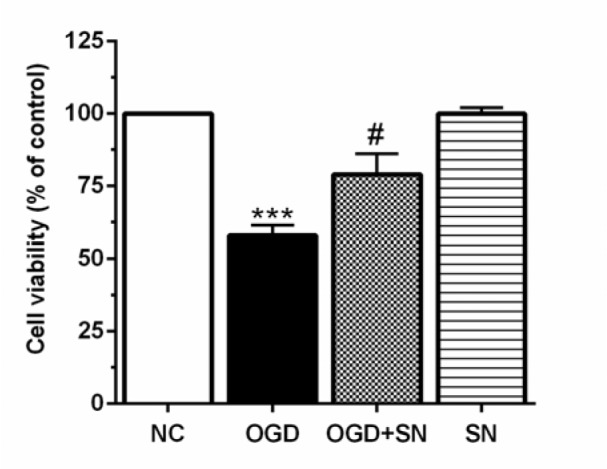
SN increased cell viability in PC12 cells-exposed to 4 h OGD. Data are presented as the percentage of control (OGD 0, normoxic normoglycemic) (n=3): ^***^p<0.001 vs. NC; ^#^p<0.05 vs. OGD [NC: normal control; OGD: oxygen and glucose deprivation (4 h); OGD-SN: oxygen and glucose deprivation (4 h) co-treated with SN (100 µm); SN: sodium nitrite (100 µm) treated].

The results of MTT showed that cell viability of OGD-exposed PC12 cells decreased markedly as compared with normal cultured cells (p<0.001; Figure 5(Fig. 5)). Administration of SN (100 µM) concomitantly with OGD onset dramatically increased cell viability (p<0.05).

For more results see the Supplementary data.

## Discussion

The findings revealed that 4 h OGD increased ROS production and Ca^2+ ^release from the ER, increased the expression of ER stress markers as well as caspases-3 and -12, while decreased cell viability. However, SN treatment reversed all changes induced by OGD insult. Recently, we showed that SN has a neuroprotective effect against OGD insult in PC12 cells through down-regulation of mitochondrial-mediated pro-apoptotic markers and up-regulation of anti-apoptotic markers (Ansari et al., 2018[6]). In the current study, we aimed to investigate whether the effect of SN-induced neuroprotective operates through modulation of ER stress markers (PERK, ATF6 and CHOP) and/or through ROS and Ca^2+^ signaling.

Cumulative evidence has confirmed that ischemia induces ROS overproduction. ROS production initiates several intracellular signaling cascades and concomitantly induces mitochondrial dysfunction and ER stress, leading to apoptotic cell death (Hildeman et al., 2003[33]; Ray et al., 2012[74]; Tajiri et al., 2004[79]). Similar to previous reports, the current results showed that OGD resulted in excessive ROS production, although SN administration attenuated ROS levels in PC12 cells exposed to OGD for 4 h and was associated with increased cell viability. Similar results have been obtained in several studies that have shown a decrease in ROS and scavenging by NO in cerebral cells, myocardial cells and gastric mucosal cells (Kwiecien et al., 2008[43]; Pluta et al., 2001[68]; Wink et al., 1993[86]; Yannopoulos et al., 2011[89]). It is believed that the neuroprotective effect of nitrite is mediated through modulation of ROS generation following ischemia and reperfusion (Ansari et al., 2018[6]; Raat et al., 2009[72]).

Prolonged ischemia disrupts the ER homeostasis that triggers cellular stress responses, including the UP response as well as the increase in p-PERK and expression of ATF6 and CHOP proteins (Feldman et al., 2005[22]; Ohoka et al., 2005[58]). Several studies have demonstrated that ischemia induces a UP response and subsequently activates PERK through phosphorylation (Badiola et al., 2011[8]; Bodalia et al., 2013[10]; Cazanave et al., 2010[14]; Hu et al., 2017[34]; Kumar et al., 2001[42]; Montie et al., 2005[51]; Vavilis et al., 2016[83]). Similarly, we found that 4 h OGD insult considerably increased ROS levels in the PC12 cells and was accompanied by an increase in ER stress markers. PERK is a type-I ER transmembrane protein with serine/threonine kinase activity in its C-terminal cytosolic domain (Bertolotti et al., 2000[9]). Once ER stress is prolonged or strong, activated PERK phosphorylates eIF2α, which in turn increases ATF6 expression (Harding et al., 1999[27], 2000[26]). Subsequently, ATF6 activates the transcription of genes involved in functional UPR and ER stress-induced apoptosis, such as CHOP (Ameri and Harris, 2008[2]; Harding et al., 2003[28]; Lange et al., 2008[44]). 

ATF6 (90 kDa) is a type-II ER transmembrane protein (Haze et al., 2001[29]). In response to the accumulation of misfolded proteins in the ER, immunoglobulin-binding protein (BiP) detaches from p90ATF6, leading to the interaction of p90ATF6 with misfolded proteins (Shen et al., 2005[76]) which ultimately results in translocation of p90ATF6 from the ER membrane to the Golgi (Chen et al., 2002[16]) and produces p50ATF6. As a result, p50ATF6 translocates to the nucleus and acts as a stimulus to increase the expression of a number of genes having protein products that participate in protein folding and protein secretion, thereby supporting the cell's effort to manage ER stress and accumulation of misfolded/unfolded proteins (Adachi et al., 2008[1]; Dorner et al., 1990[19]; Gething and Sambrook, 1992[24]; Haze et al., 1999[30]; Healy et al., 2009[31]; Li et al., 2000[45]; Parmar and Schroder, 2012[64]; Yamamoto et al., 2007[88]; Yoshida et al., 2000[91], 2001[90]). In the present study, a strong decrease in ATF6 expression was observed. We used p90ATF6 antibody for determining the activity of this marker by Western blotting. The results showed that, in the OGD condition, the quantity of p90ATF6 decreased, suggesting that OGD increases the cleavage of p90ATF6 to p50ATF6. Nevertheless, in our study, SN reversed these changes, suggesting that SN attenuated the protein entry from the ER to the Golgi apparatus and restored neuronal homeostasis during OGD-induced ER stress. 

CHOP is a downstream product of the PERK-eIF2α-ATF6 pathway which induces apoptosis under ER stress through down-regulation of anti-apoptotic factor such as Bcl-2, up-regulation of pro-apoptotic factors and ROS production (Oida et al., 2008[59]; Tajiri et al., 2004[79]; Zhao et al., 2005)[92]. It has been shown that CHOP suppression protects astrocytes from OGD injury, whereas CHOP overexpression results in astrocyte apoptosis (Osada et al., 2010[60]). Our results demonstrate that 4 h OGD insult up-regulated the expression of CHOP, possibly through the PERK-eIF2α-ATF6 pathways. Moreover, overexpression of CHOP was associated with an increase in the cleavage of caspase-12 and caspase-3. According to previous studies, the caspase-4 dependent pathway and mitochondrial-dependent apoptotic pathways also are activated by ER stress, which leads to activation of caspases-12 and caspase-9 to activate caspase-3, an initiator of the apoptosis (Arduíno et al., 2009[7]; Oyadomari and Mori, 2004[62]).

SN treatment significantly decreased the expression of CHOP in PC12 cells. Our findings suggest that the decrease in phosphorylation of PERK, as well as down-regulation of CHOP by SN treatment, may change the balance in favor of cell survival. SN treatment through down-regulation of the extrinsic apoptotic pathway, caspase-12 and -3 increased resistance to OGD-induced apoptosis. Several investigations also have confirmed that NO directly and indirectly attenuated caspase activity in cells (Ansari et al., 2018[6]; Kim et al., 1997[41], 2002[40]).

Nitrite, a NO donor, can be reduced to NO under low oxygen and pH conditions. Studies have shown that, after OGD, the fall in mitochondrial pH provides appropriate conditions for the reduction of nitrite to NO (Butler and Ridd, 2004[11]). Nitrite-derived NO can prevent neuronal nuclear translocation of NF-κB, an apoptosis-related transcription factor, resulting in the down-regulation of NF-κB target genes that are generally involved in inflammatory response and apoptotic pathways (Miller and Megson, 2007[49]; Mo et al., 2016[50]). 

ER, the major internal Ca^2+^ storage organelle, acts as a Ca^2+^ buffer and controls Ca^2+^-dependent signaling in the cytosol (Morishima et al., 2002[53]). Activation of the UP response and overproduction of ROS resulting from OGD disrupted the intracellular Ca^2+ ^homeostasis, leading to activation of calpains. Calpains, in turn, activates the caspases, particularly pro-caspase-12, promoting cell death (Nakagawa and Yuan, 2000[55]). The results also demonstrate that OGD-induced ROS production induced ER stress markers as well as disrupting the ER membrane and triggered Ca^2+^ release from the ER in PC12 injured cells, causing cleavage of procaspase-12 and procaspase-3 and cell death, as confirmed by the low cell viability. It should be noted that co-treatment of SN with OGD insult decreased Ca^2+^ release. The inhibitory effects of NO on ER Ca^2+^ release have been demonstrated in skeletal muscle fibers, myocytes and small mesenteric arteries. NO can also modulate agonist-evoked intracellular Ca^2+^ release in neurosecretory PC12 cells (Clementi et al., 1995[17]; Pouvreau and Jacquemond, 2005[69]; Pucovsky et al., 2002[70]). 

Our findings reveal that treatment with 100 µmol SN attenuated OGD-induced ROS production, Ca^2+^ release and subsequently decreased ER stress-related markers such as p-PERK, ATF6, and CHOP expression as well as caspase-12 and caspase-3 activation. These results were confirmed by the increased cell viability as indicated by MTT assay. Some studies have reported contradictory results. For instance, it has been reported that a 1 mM dose of sodium nitroprusside induced ER stress and increased expression of CHOP as well as apoptosis in the chondrocytes (Takada et al., 2013[80]). Oyadomari et al. (2001[63]) found that 0.5 mM S-nitroso-N-acetyl-D,L-penicillamine (SNAP) induces ER stress and CHOP expression in pancreatic β cells. Uchiyama et al. (2002[82]) also reported that a high concentration (1 mM) of diethylenetriamine NO or SNAP provoked both apoptosis and necrosis through activation of caspase-3 in the cardiomyocytes of neonatal rats. Furthermore, NO increases the activity of ryanodine receptors 1 and 2 (RyR1 and RyR2), a class of intracellular Ca^2+ ^channels, in excitable tissues such as muscles and neurons through S-nitrosylation (Xu et al., 1998[87]). These discrepancies in the protective or toxic effects of NO donors are possibly due to differences in NO donor type, treatment concentration or doses, time of release, cell type and treatment conditions, as many researchers have reported that the dose of the NO donor plays an important role in its cytoprotective effect(Khan et al., 2005[39], 2006[38]; Takuma et al., 2002[81]; Wedgwood and Black, 2003[85]).

**Figure 6:**
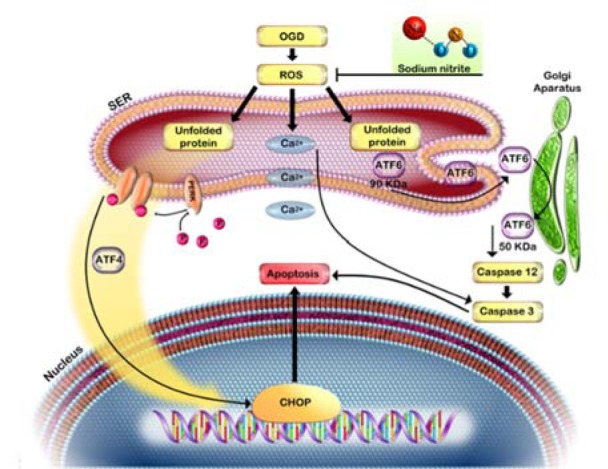
Oxygen and glucose deprivation induced ROS overproduction, leading to ER stress and cell death

Overall, our findings demonstrated that OGD decreased cell viability and induced cell death in PC12 cells which were mediated by activation of ROS generation and induction of ER stress. However, SN as a NO donor at a low concentration (100 µM), by reducing ROS generation, decreased expression of ER stress markers and increased cell viability in OGD injured PC12 (Figure 6(Fig. 6)). Our findings may provide a rationale for further research and the development of the use of SN as a NO donor against ischemic insult.

## Notes

Mohammad Hassan Khadem Ansari and Pouran Karimi (Neurosciences Research Center (NSRC), Tabriz University of Medical Sciences, Tabriz, Iran; E-mail: karimip@tbzmed.ac.ir) contributed equally as corresponding authors.

## Conflict of interest

All authors declare no conflict of interest.

## Ethical approval

This article does not contain any studies with human participants or animals performed by any of the authors.

## Supplementary Material

Supplementary data
