# Supplementary material for: Neuroprotective mechanism of low-dose sodium nitrite in oxygen-glucose deprivation model of cerebral ischemic stroke in PC12 cells
Source: EXCLI J. 2019 Apr 8;18:229–42. doi: 10.17179/excli2018-1947 (PMC6558507; doi:10.17179/excli2018-1947)
Supplement: Supplementary data [file EXCLI-18-229-s-001.pdf]

EXCLI Journal 2019;18:229-242 – ISSN 1611-2156
Received: November 27, 2018, accepted: March 26, 2019, published: April 08, 2019

Supplementary data to:

NEUROPROTECTIVE MECHANISM OF LOW-DOSE SODIUM NITRITE IN OXYGEN-GLUCOSE DEPRIVATION MODEL OF CEREBRAL ISCHEMIC STROKE IN PC12 CELLS

Nader Shakib[1], Mohammad Hassan Khadem Ansari[1*], Pouran Karimi [2]*, Yousef Rasmi[1]

[1] Department of Biochemistry, Faculty of Medicine, Urmia University of Medical Sciences, Urmia, Iran
[2] Neurosciences Research Center (NSRC), Tabriz University of Medical Sciences, Tabriz, Iran

* Corresponding authors: Dr. Mohammad Hassan Khadem Ansari, Department of Biochemistry, Faculty of Medicine, Urmia University of Medical Sciences, Urmia, Iran; E-mail: ansari_mh@umsu.ac.ir;
Dr. Pouran Karimi, Neurosciences Research Center (NSRC), Tabriz University of Medical Sciences, Tabriz, Iran; E-mail: karimip@tbzmed.ac.ir

http://dx.doi.org/10.17179/excli2018-1947

This is an Open Access article distributed under the terms of the Creative Commons Attribution License (http://creativecommons.org/licenses/by/4.0/).

EXCLI Journal 2019;18:229-242 – ISSN 1611-2156
Received: November 27, 2018, accepted: March 26, 2019, published: April 08, 2019

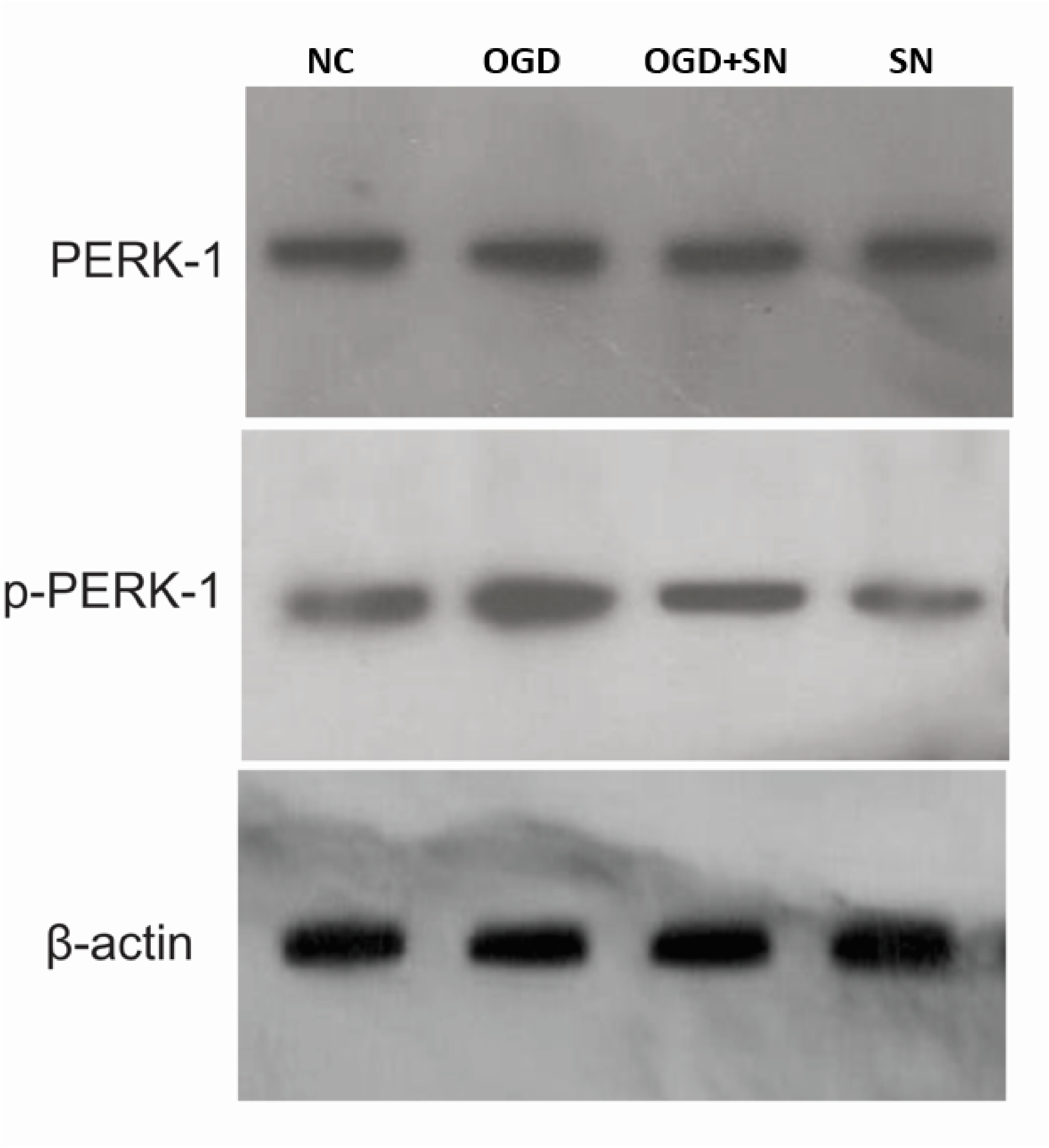

Figure 1: Immunoblotting images of expression of p-PERK, PERK, ATF6, CHOP, and β-actin proteins in three independent tests

NC: normal control; OGD: oxygen and glucose deprivation (4 h); OGD-SN: oxygen and glucose deprivation (4 h) co-treated with SN (100 µm); SN: sodium nitrite (100 µm) treated

EXCLI Journal 2019;18:229-242 – ISSN 1611-2156
Received: November 27, 2018, accepted: March 26, 2019, published: April 08, 2019

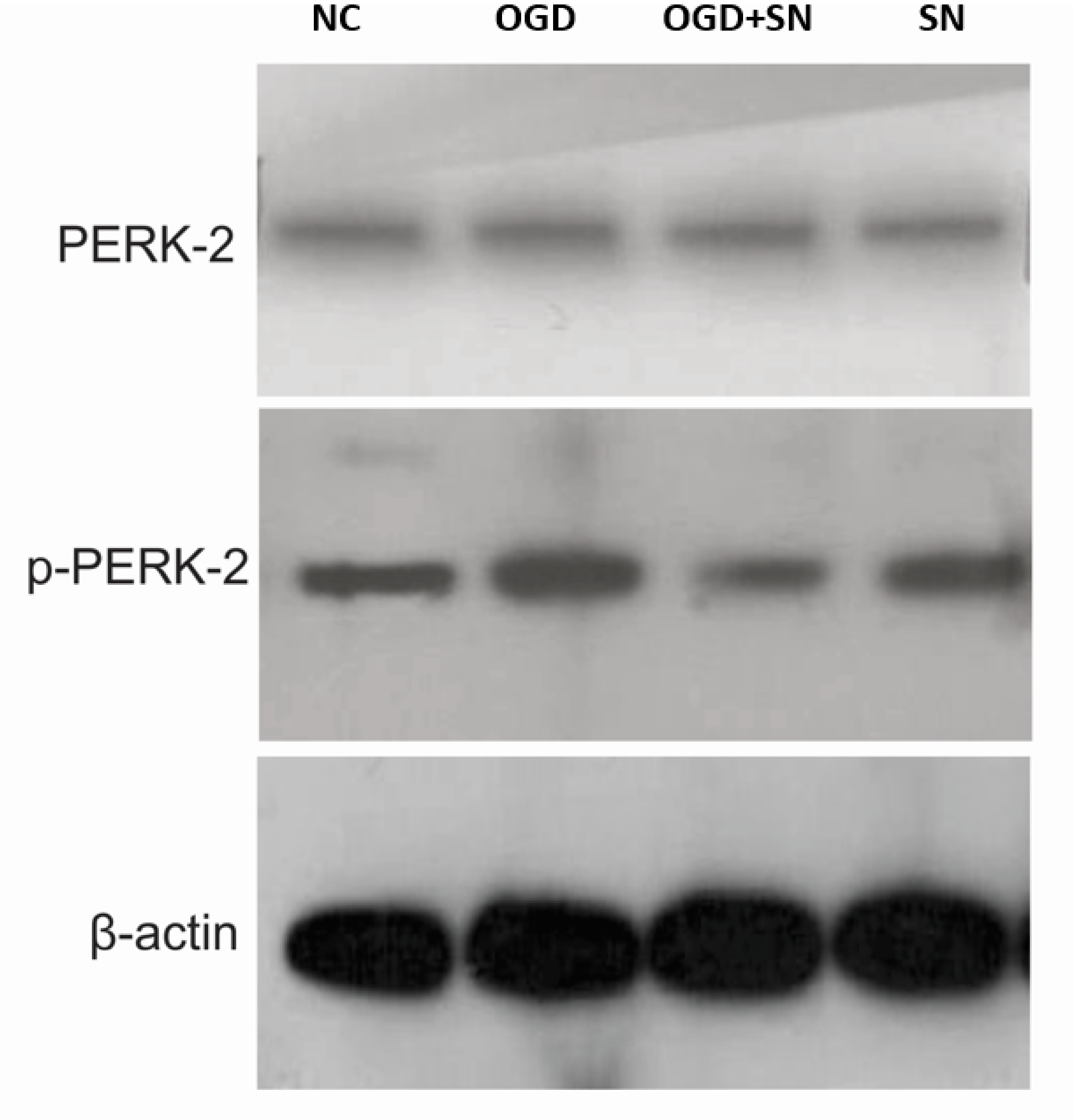

Figure 1 (cont.): Immunoblotting images of expression of p-PERK, PERK, ATF6, CHOP, and β-actin proteins in three independent tests

EXCLI Journal 2019;18:229-242 – ISSN 1611-2156
Received: November 27, 2018, accepted: March 26, 2019, published: April 08, 2019

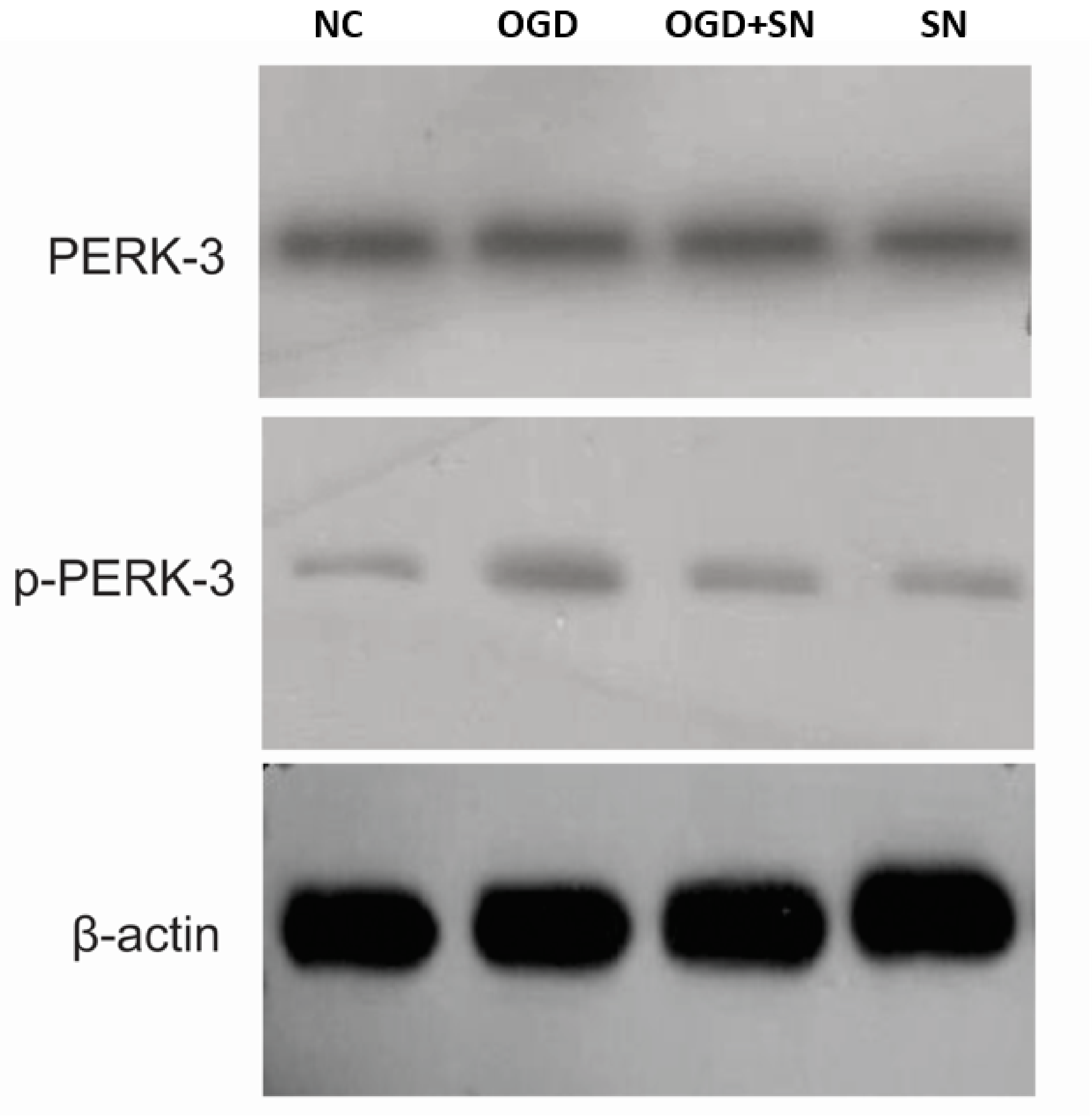

Figure 1 (cont.): Immunoblotting images of expression of p-PERK, PERK, ATF6, CHOP, and β-actin proteins in three independent tests

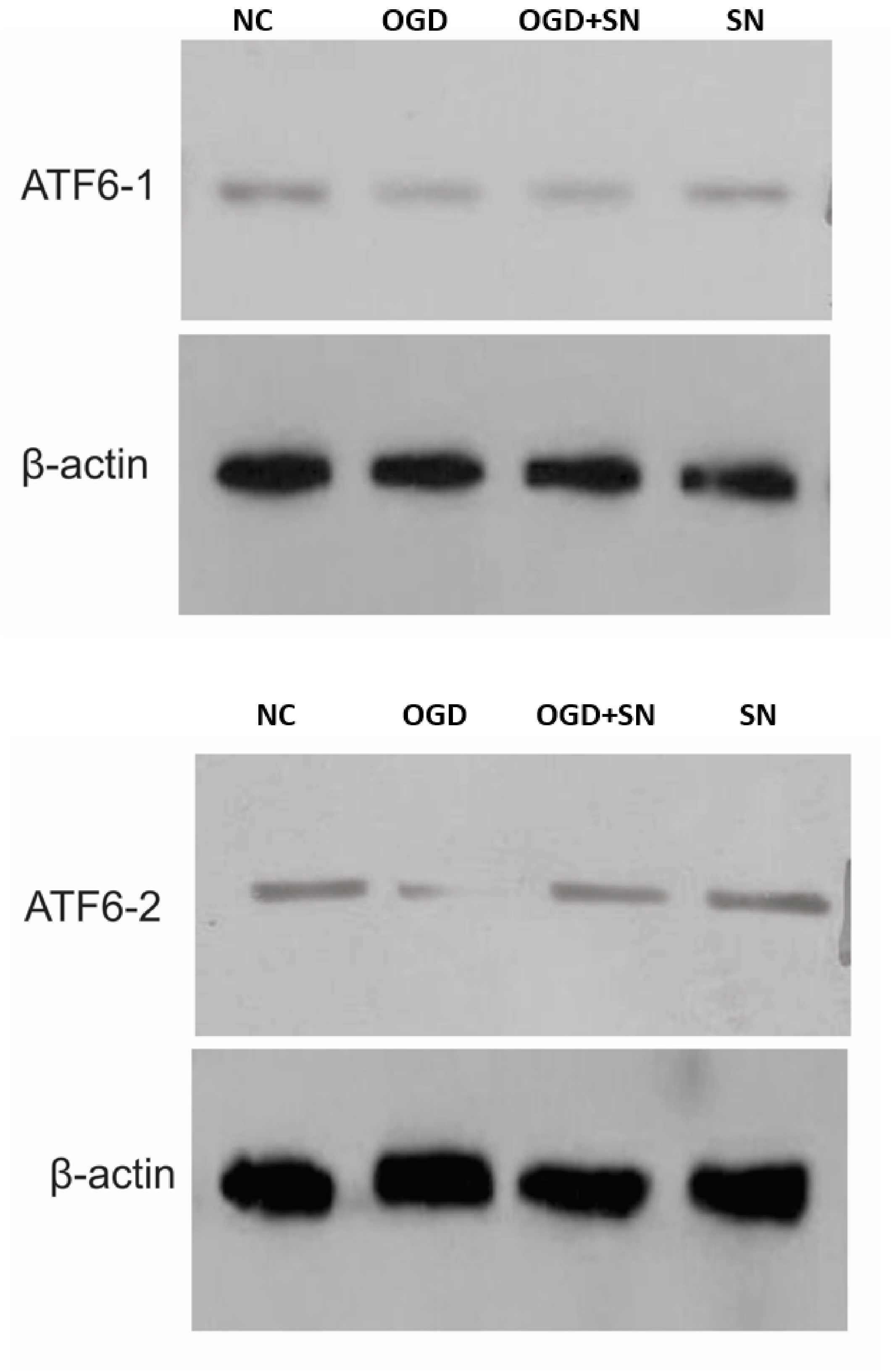

Figure 1 (cont.): Immunoblotting images of expression of p-PERK, PERK, ATF6, CHOP, and β-actin proteins in three independent tests

EXCLI Journal 2019;18:229-242 – ISSN 1611-2156
Received: November 27, 2018, accepted: March 26, 2019, published: April 08, 2019

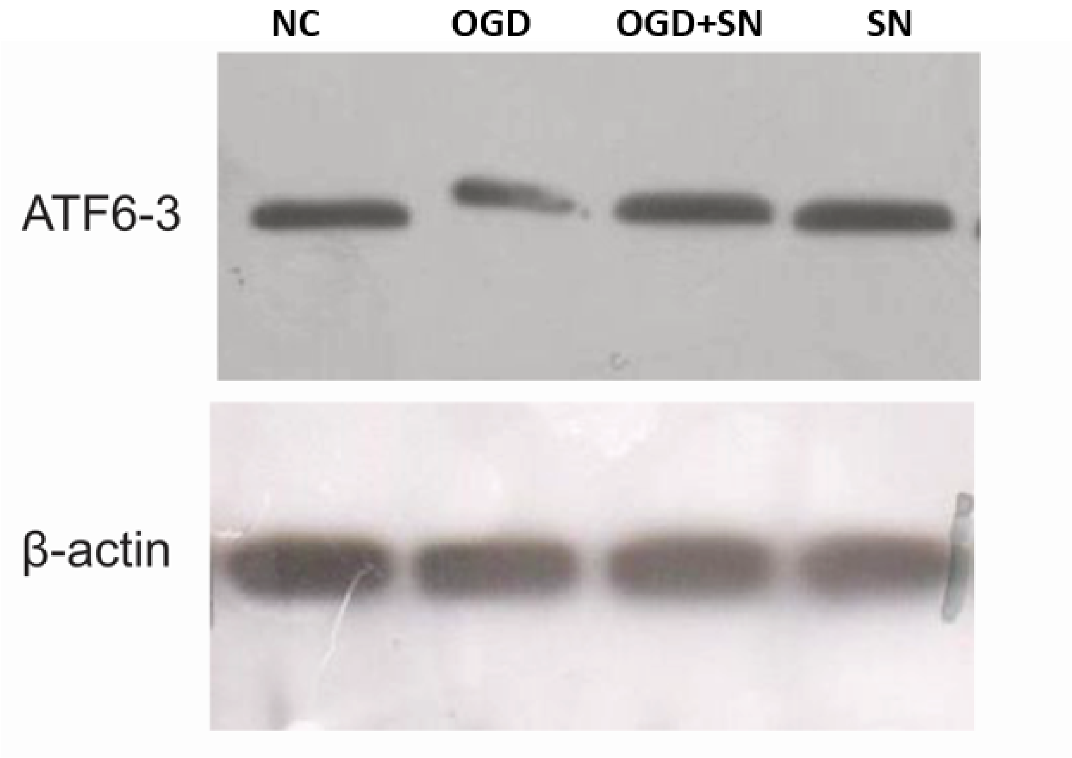

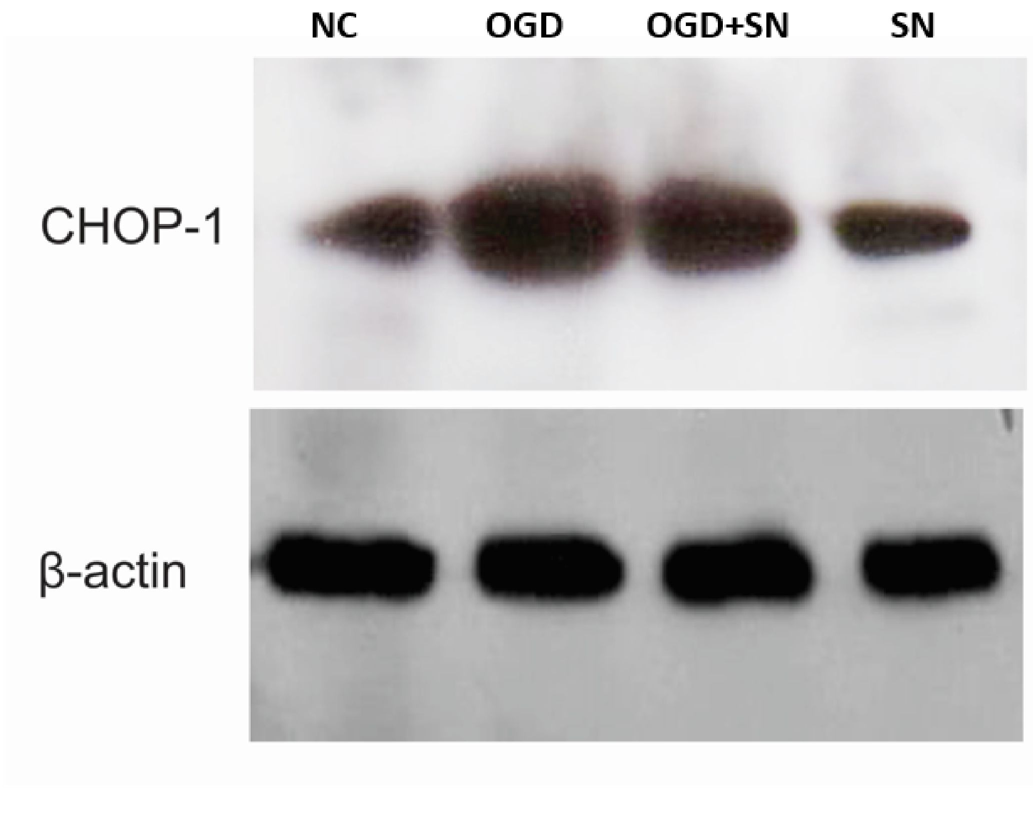

Figure 1 (cont.): Immunoblotting images of expression of p-PERK, PERK, ATF6, CHOP, and β-actin proteins in three independent tests

EXCLI Journal 2019;18:229-242 – ISSN 1611-2156
Received: November 27, 2018, accepted: March 26, 2019, published: April 08, 2019

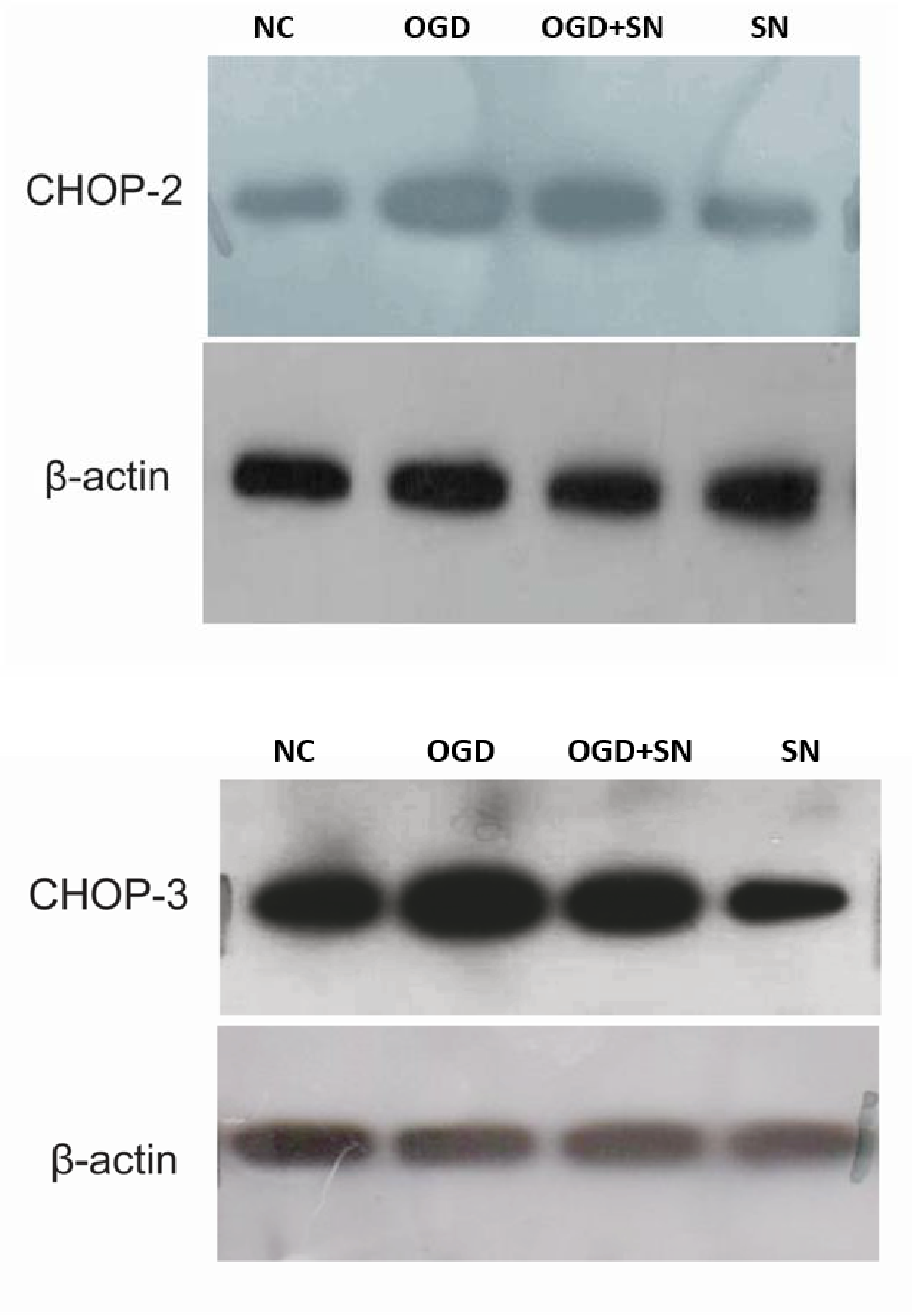

Figure 1 (cont.): Immunoblotting images of expression of p-PERK, PERK, ATF6, CHOP, and β-actin proteins in three independent tests

EXCLI Journal 2019;18:229-242 – ISSN 1611-2156
Received: November 27, 2018, accepted: March 26, 2019, published: April 08, 2019

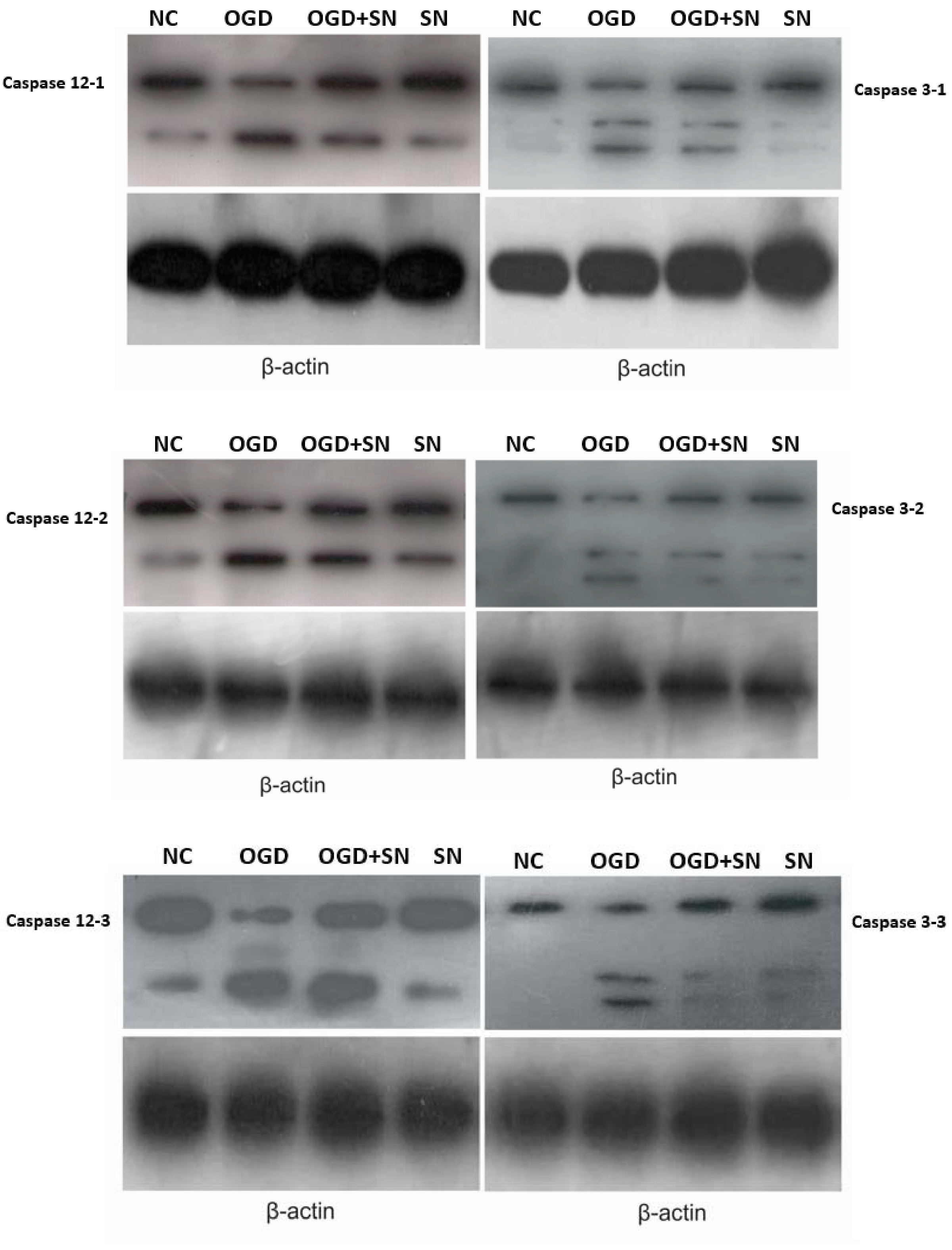

Figure 2: Immunoblotting images of caspase-12, caspase-3, and β-actin in three independent tests

NC: normal control; OGD: oxygen and glucose deprivation (4 h); OGD-SN: oxygen and glucose deprivation (4 h) co-treated with SN (100 µm); SN: sodium nitrite (100 µm) treated
